# Characterisation of a G2P[4] Rotavirus Outbreak in Western Australia, Predominantly Impacting Aboriginal Children

**DOI:** 10.3390/pathogens10030350

**Published:** 2021-03-16

**Authors:** Celeste M. Donato, Nevada Pingault, Elena Demosthenous, Susie Roczo-Farkas, Julie E. Bines

**Affiliations:** 1 Enteric Diseases Group, Murdoch Children’s Research Institute, Parkville 3052, Australia; ahahahaha@gmai.com (E.D.); susie.roczofarkas@mcri.edu.au (S.R.-F.); jebines@unimelb.edu.au (J.E.B.); 2 Department of Paediatrics, The University of Melbourne, Parkville 3010, Australia; 3 Department of Microbiology, Biomedicine Discovery Institute, Monash University, Clayton 3800, Australia; 4 Department of Health Western Australia, Communicable Disease Control Directorate, Perth 6004, Australia; nevada.pingault@health.wa.gov.au; 5 Department of Gastroenterology and Clinical Nutrition, Royal Children’s Hospital, Parkville 3052, Australia

**Keywords:** rotavirus, outbreak, Aboriginal, Indigenous, G2P[4], gastroenteritis, Western Australia, whole genome sequencing, vaccine

## Abstract

In May, 2017, an outbreak of rotavirus gastroenteritis was reported that predominantly impacted Aboriginal children ≤4 years of age in the Kimberley region of Western Australia. G2P[4] was identified as the dominant genotype circulating during this period and polyacrylamide gel electrophoresis revealed the majority of samples exhibited a conserved electropherotype. Full genome sequencing was performed on representative samples that exhibited the archetypal DS-1-like genome constellation: G2-P[4]-I2-R2-C2-M2-A2-N2-T2-E2-H2 and phylogenetic analysis revealed all genes of the outbreak samples were closely related to contemporary Japanese G2P[4] samples. The outbreak samples consistently fell within conserved sub-clades comprised of Hungarian and Australian G2P[4] samples from 2010. The 2017 outbreak variant was not closely related to G2P[4] variants associated with prior outbreaks in Aboriginal communities in the Northern Territory. When compared to the G2 component of the RotaTeq vaccine, the outbreak variant exhibited mutations in known antigenic regions; however, these mutations are frequently observed in contemporary G2P[4] strains. Despite the level of vaccine coverage achieved in Australia, outbreaks continue to occur in vaccinated populations, which pose challenges to regional areas and remote communities. Continued surveillance and characterisation of emerging variants are imperative to ensure the ongoing success of the rotavirus vaccination program in Australia.

## 1. Introduction

Group A rotaviruses, belonging to the Reoviridae virus family, remain one of the main aetiological agents of acute gastroenteritis in infants and young children worldwide, estimated to have caused 128,500 deaths and 258,173,300 episodes of diarrhea among children <5 years of age in 2016 [1]. The substantial decrease in the global burden of rotavirus disease over the last decade can be attributed to varied public health measures, such as improved sanitation, as well as the inclusion of rotavirus vaccines into the National Immunisation Programs (NIPs) of over 100 countries worldwide [2]. In Australia, the live-attenuated vaccines Rotarix^®^ (monovalent, human G1P[8] strain) and RotaTeq® (pentavalent, human-bovine reassortant vaccine comprising G1P[5], G2P[5], G3P[5], G4P[5], and G6P[8] strains) were introduced into the NIP in mid-2007, with a state-based vaccine selection method in place up until mid-2017, after which a national tender process was initiated, with all states and territories now using Rotarix [3,4].

Group A rotavirus strains are classified into G and P genotypes based on the outer capsid proteins VP7 and VP4, respectively. To date, 36 G types and 51 P types have been characterised from humans and varied animal species [5]. The most prevalent genotypes in humans are G1, G2, G3, G4, G9, and G12, in combination with P[4], P[6], and P[8] [6,7]. A whole genome classification nomenclature has been developed to describe the genome constellation of strains; Gx-P[x]-Ix-Rx-Cx-Mx-Ax-Nx-Tx-Ex-Hx, denoting the VP7-VP4-VP6-VP1-VP2-VP3-NSP1-NSP2-NSP3-NSP4-NSP5/6 genes, with x referring to the various recognised genotypes for each gene. There are three major genotype constellations: Wa-like (G1-P[8]-I1-R1-C1-M1-A1-N1-T1-E1-H1), DS-1-like (G2-P[4]-I2-R2-C2-M2-A2-N2-T2-E2-H2), and AU-1-like (G3-P[9]-I3-R3-C3-M3-A3-N3-T3-E3-H3) [8].

Western Australia (WA) is the largest state in Australia and is sparsely populated, with 80% of the 2.5 million residents residing in the capital city of Perth. Approximately 4% of the WA population identify as Indigenous (hereafter respectfully referred to as Aboriginal to recognise that Aboriginal people are the original inhabitants of WA), the proportion is higher outside Perth [9]. The Kimberley (KIMB) is a remote region that encompasses an area of 421,451 square kilometres. In 2016, the population was 36,392, and 45% of the region’s population identified as Aboriginal, living in towns and communities of varying sizes. The KIMB region has a younger population compared to other regions of WA, with a higher percentage of children aged 0–14 years (25%) [10]. For the period 2011–2015, the enteric disease notification rate (salmonellosis, cryptosporidiosis, rotavirus, campylobacteriosis, and shigellosis) for children in the KIMB region was 5.2 times higher than for all children in WA, with rotavirus accounting for 5% of notifications. For all enteric infection notifications, the rate for Aboriginal children was 2.4 times the non-Aboriginal rate [10].

Rotavirus became a notifiable disease in WA from July 2006 [11]. The Communicable Disease Control Directorate (CDCD) and Public Health Units (PHUs) in the Department of Health WA (WA Health) investigate clusters and outbreaks of rotavirus. Initially, Rotarix was used in WA, from July 2007 to February 2009, then vaccine selection was changed to RotaTeq. In July 2017, the rotavirus vaccine used in WA reverted back to Rotarix [12,13]. In 2017, the estimated vaccine coverage in eligible children <12 months of age was 83.5% in Aboriginal children nationally and 89.5% in non-Aboriginal children [14]. The vaccine coverage for WA was 81.5% in 2015, the most recent data available, compared to a national coverage of 85.4% [15].

Sporadic community-wide rotavirus outbreaks have occurred in different states and territories around Australia. Outbreaks due to G2P[4] strains occurred in Perth (1993), Melbourne (1994), and Sydney (2001) [16,17]. Widespread outbreaks impacting remote communities in the Northern Territory have occurred due to G2P[4] strains in 1993, 1999, 2004, and 2009 [18,19,20,21,22]. Outbreaks due to G2P[4] strains were reported in 2010 in South Australia and Western Australia [23]. An outbreak caused by G2P[4] occurred in New South Wales in 2012, predominantly impacting children aged 5–9 years [24]. In 2017, multiple G2P[4] outbreaks were reported in the Northern Territory, South Australia, and Western Australia [3].

The aim of this study was to describe the epidemiology and burden of disease during an outbreak of rotavirus in the metropolitan (METRO) region of Perth and the remote Kimberley (KIMB) region of WA in 2017. Whole genome sequencing was performed to characterise the rotavirus strain circulating during this outbreak and place it in the context of global strains.

## 2. Results

### 2.1. Descriptive Epidemiology

**Figure 1:**
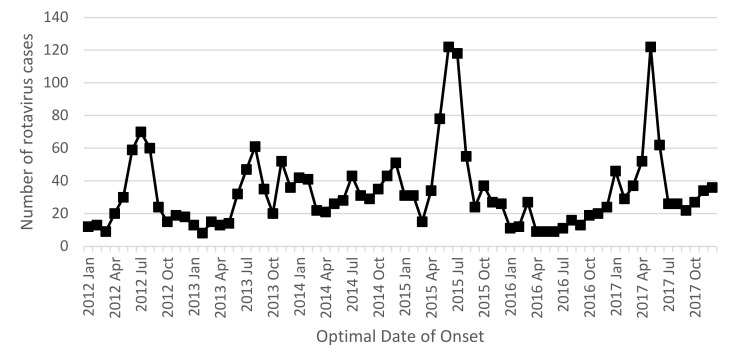
Monthly rotavirus notification rates between January 2012 and November 2017.

In 2017, there were 519 notified cases of rotavirus infection in WA (19.1 cases per 100,000 population), making rotavirus the third most commonly notified enteric infection in WA. A marked increase in rotavirus notifications was noted in the second quarter of 2017 (April/May/June, 2Q17), with 236 cases, compared to the five-year second quarter average (2012–2016) of 100.8 cases (Figure 1).

**Figure 2:**
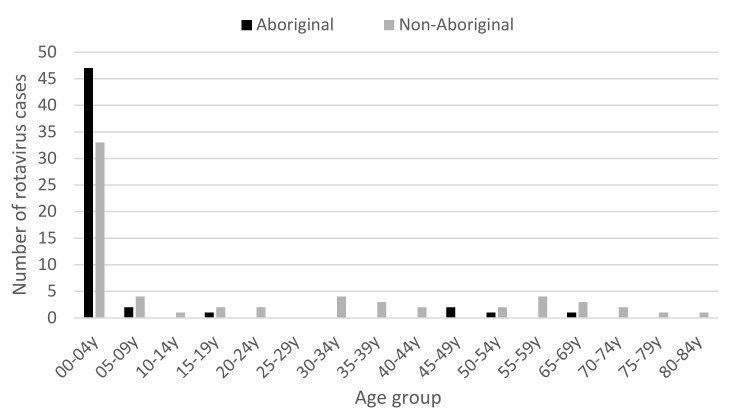
Distribution of the number of rotavirus cases in Western Australia in May, 2017 by age (years) and Indigenous status.

**Figure 3:**
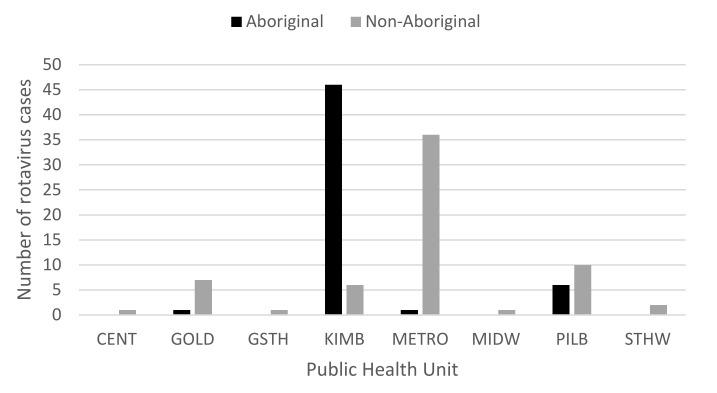
Distribution of the number of rotavirus cases from May, 2017, all ages, by Indigenous status and Public Health Unit (PHU) boundaries, reflecting WA Health administrative regions: Central/Wheatbelt (CENT), Goldfields (GOLD), Great Southern (GSTH), Kimberley (KIMB), Metropolitan Perth (METRO), Midwest (MIDW), Pilbara (PILB), and South West (STHW).

**Figure 4:**
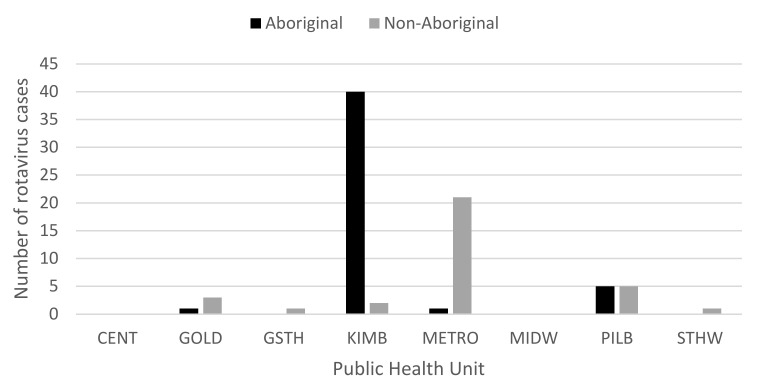
Distribution of rotavirus cases in May, 2017, aged ≤4 years, by Aboriginal status and Public Health Unit (PHU) boundaries, reflecting WA Health administrative regions: Central/Wheatbelt (CENT), Goldfields (GOLD), Great Southern (GSTH), Kimberley (KIMB), Metropolitan Perth (METRO), Midwest (MIDW), Pilbara (PILB), and South West (STHW).

Within the 2Q17, the highest number of cases was seen in May (*n* = 122), compared to 52 cases in April and 62 cases in June. Of the 122 cases in May, 80 were aged ≤4 years (Figure 2), with the majority of cases aged <1 year (*n* = 26) and 1 year (*n* = 31). While cases were seen in all PHUs, Aboriginal people from the KIMB (*n* = 46) region and non-Aboriginal people from the metropolitan (METRO) region (*n* = 36) were the two most affected groups (Figure 3). Examining children ≤4 years, Indigenous status, and PHU more closely, Aboriginal children from the KIMB region were disproportionally represented (40/80 cases) (Figure 4).

Vaccination status was known for 97% of the total May cases (118/122). Of these, 41% were fully vaccinated (48/118), 24% were partially vaccinated (29/118), and 35% were not vaccinated (41/118) (Table 1). Only five of the unvaccinated cases were eligible to have been vaccinated, with the remaining 36 cases ineligible due to age.

Hospitalisation status was known for 78% of cases (95/122). For those with known hospitalisation status, 38% (36/95) were hospitalised as a result of their infection, of which 64% were Aboriginal people (23/36) and 36% were non-Aboriginal people (13/36). Children aged ≤4 years represented 86% of hospitalisations (31/36), of which Aboriginal children accounted for 71% (22/31). Of the hospitalised cases, 39% were fully vaccinated (14/36) and 36% were partially vaccinated (13/36). A further 22% were not vaccinated (8/36), five of which were ineligible for vaccination due to age. Vaccination status was unknown for one case.

### 2.2. Genotyping

Cases were designated to the month of May based on the optimal date of onset (ODOO). Of the 122 cases from the month of May, a stool sample was available for 70 and were sent for genotype analysis at the National Rotavirus Reference Centre (NRRC), Murdoch Children’s Research Institute in Melbourne, Australia. The predominant genotype identified was G2P[4] (94%, 66/70) (Table 2), with the majority of G2P[4] cases in the KIMB region (61%, 40/66). In the KIMB region, Aboriginal people were disproportionately represented, accounting for 90% of cases (36/40) (Table 2).

### 2.3. Vaccination and Hospitalisation Status of Genotyped Cases

Rotavirus vaccination information was available for 94% (66/70) of cases notified in May with genotyping results (Table 1). Of these, 53% (35/66) were fully vaccinated, 20% (13/66) were partially vaccinated, 4% (3/66) were not vaccinated but were eligible based on age, and 23% (15/66) were not vaccinated due to age (Table 1). Three quarters of G2P[4] cases were either fully or partially vaccinated (47/62). The majority of cases that were partially or fully vaccinated had received only the RotaTeq vaccine. Two fully vaccinated and one partially vaccinated case had received the Rotarix vaccine. Two fully vaccinated cases had received a combination of Rotarix and RotaTeq vaccines. Almost all Aboriginal cases had a known vaccination status (40/41); 60% (24/40) were fully vaccinated, 30% (12/40) were partially vaccinated, and 10% (4/20) were not vaccinated. Of the non-Aboriginal cases with known vaccination status (26/29), 42% (11/26) were fully vaccinated, 4% (1/26) were partially vaccinated, and 54% (14/26) were not vaccinated.

Hospitalisation status was known for 86% of the genotyped cases in May (60/70), with 19 cases hospitalised. Of these, 47% were fully vaccinated (9/19), 26% were partially vaccinated (5/19), 16% were not vaccinated (3/19), and vaccination status was unknown for two cases (10%).

### 2.4. Sequence Analysis of G2P[4] Samples

A total of 38 G2P[4] samples were analysed using polyacrylamide gel electrophoresis to visualise the electropherotype pattern. The majority of samples had a highly similar electropherotype, indicating that a relatively conserved strain was circulating during the outbreak (data not shown).

Three samples were selected for whole genome sequencing, which were representative of the dominant electropherotype: RVA/Human-wt/AUS/WAPC2769/2017/G2P[4] (1-year-old, fully vaccinated, KIMB), RVA/Human-wt/AUS/WAPC2784/2017/G2P[4] (2-year-old, fully vaccinated child, METRO), and RVA/Human-wt/AUS/WAPC2824/2017/G2P[4] (3-year-old, fully vaccinated KIMB). The three samples exhibited the archetypal DS-1-like genome constellation: G2-P[4]-I2-R2-C2-M2-A2-N2-T2-E2-H2.

The 11 genes of each sample were successfully sequenced, with the exception of the VP2 gene of RVA/Human-wt/AUS/WAPC2769/2017/G2P[4] and RVA/Human-wt/AUS/WAPC2824/2017/G2P[4] for which only 69.7–70.3% of the open reading frame (ORF) could be determined. The sample volumes were exhausted, attempting to resolve the approximate 800-base pair (bp) region at the 3′ prime end of the gene without success.

The coding regions of each gene of RVA/Human-wt/AUS/WAPC2784/2017/G2P[4] and RVA/Human-wt/AUS/WAPC2824/2017/G2P[4] were highly conserved, with RVA/Human-wt/AUS/WAPC2769/2017/G2P[4] displaying some minor variability: VP1 (99.81–99.94% nucleotide (nt) and 99.82–99.91% amino acid (aa) similarity), VP2 (99.84–100% nt and 99.84–100% aa similarity), VP3 (99.84–99.92% nt and 99.88–100% aa similarity), VP4 (99.79–99.91% nt and 99.61–99.87% aa similarity), VP6 (99.92–100% nt and 100% aa similarity), VP7 (99.89–100% nt 99.69–100% aa similarity), NSP1 (99.86–99.93% nt and 100% aa similarity), NSP2 (99.90–100% nt and 100% aa similarity), NSP3 (99.47–99.79% nt and 99.39–99.68% aa similarity), NSP4 (99.62–99.81% nt and 99.43–100% aa similarity), and NSP5/6 (99.86–99.93% nt and 100% aa similarity).

### 2.5. Phylogenetic Analysis

**Figure 5:**
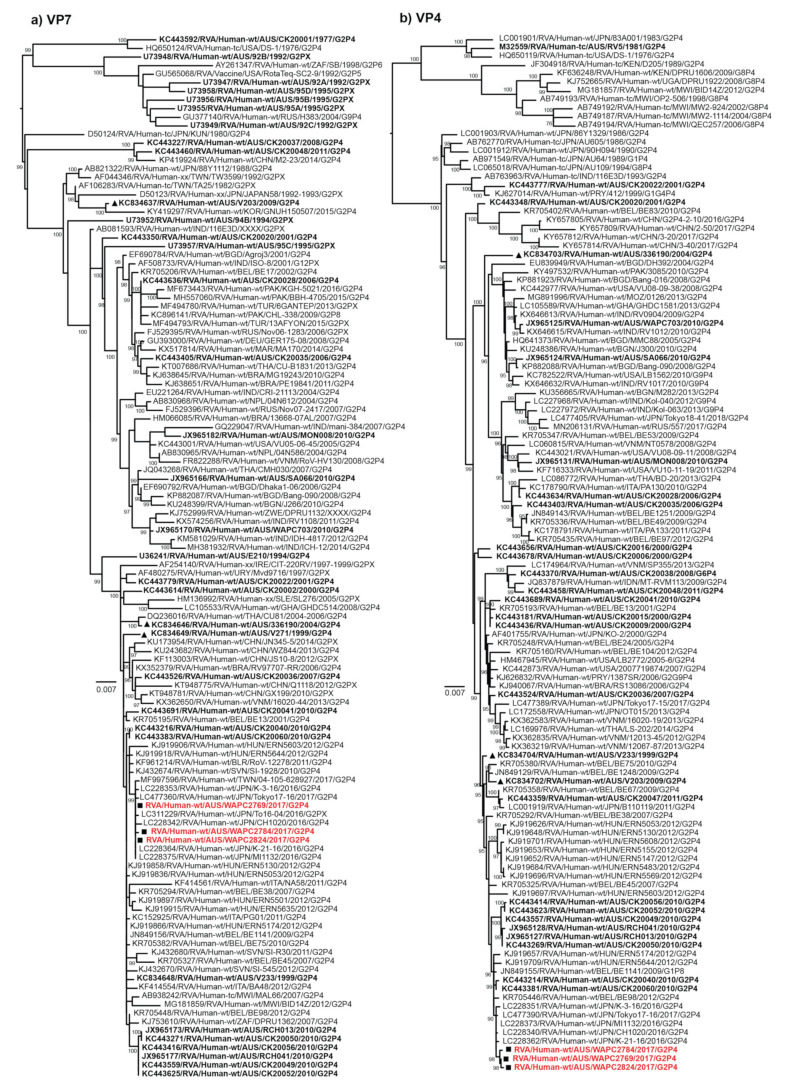

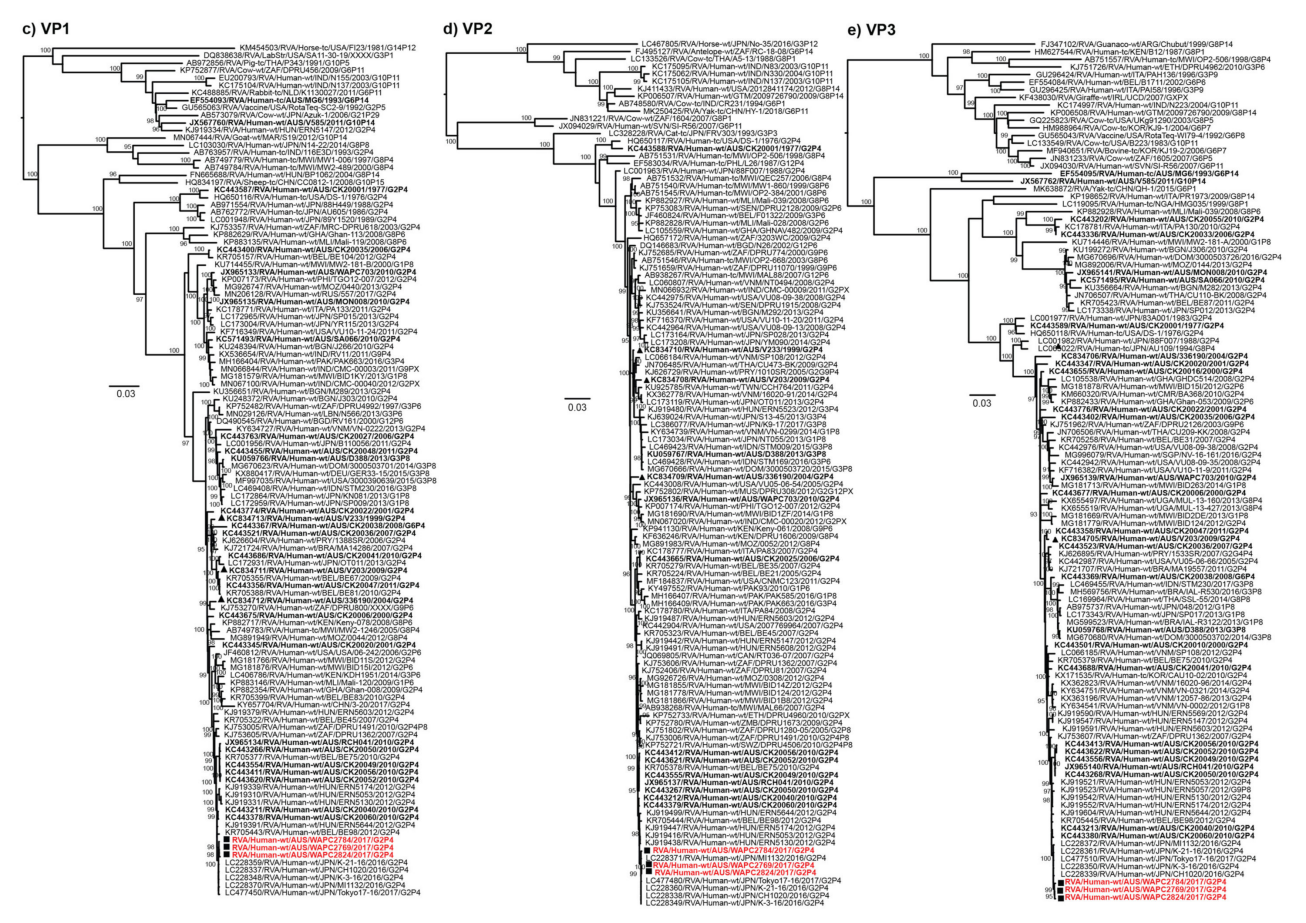

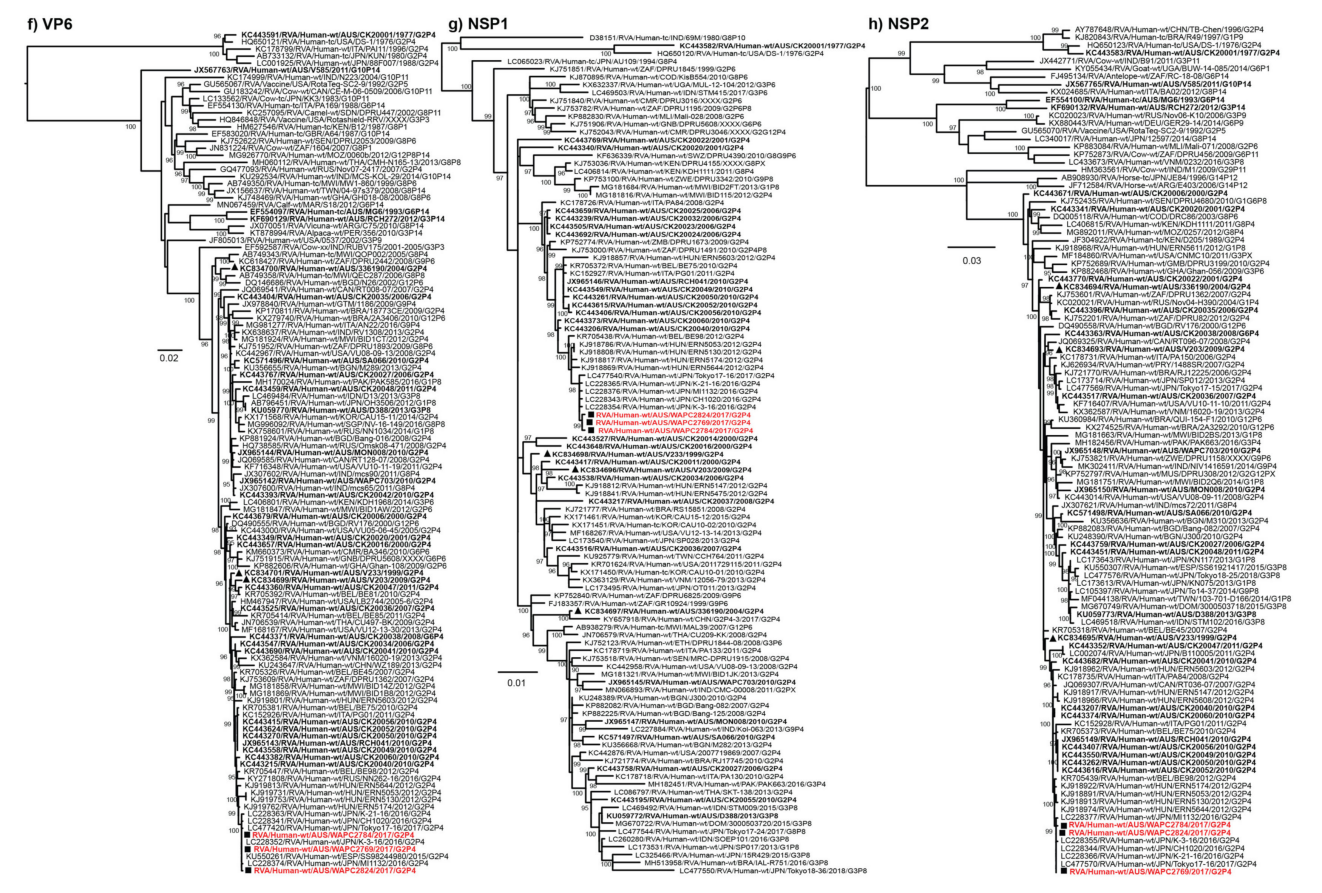

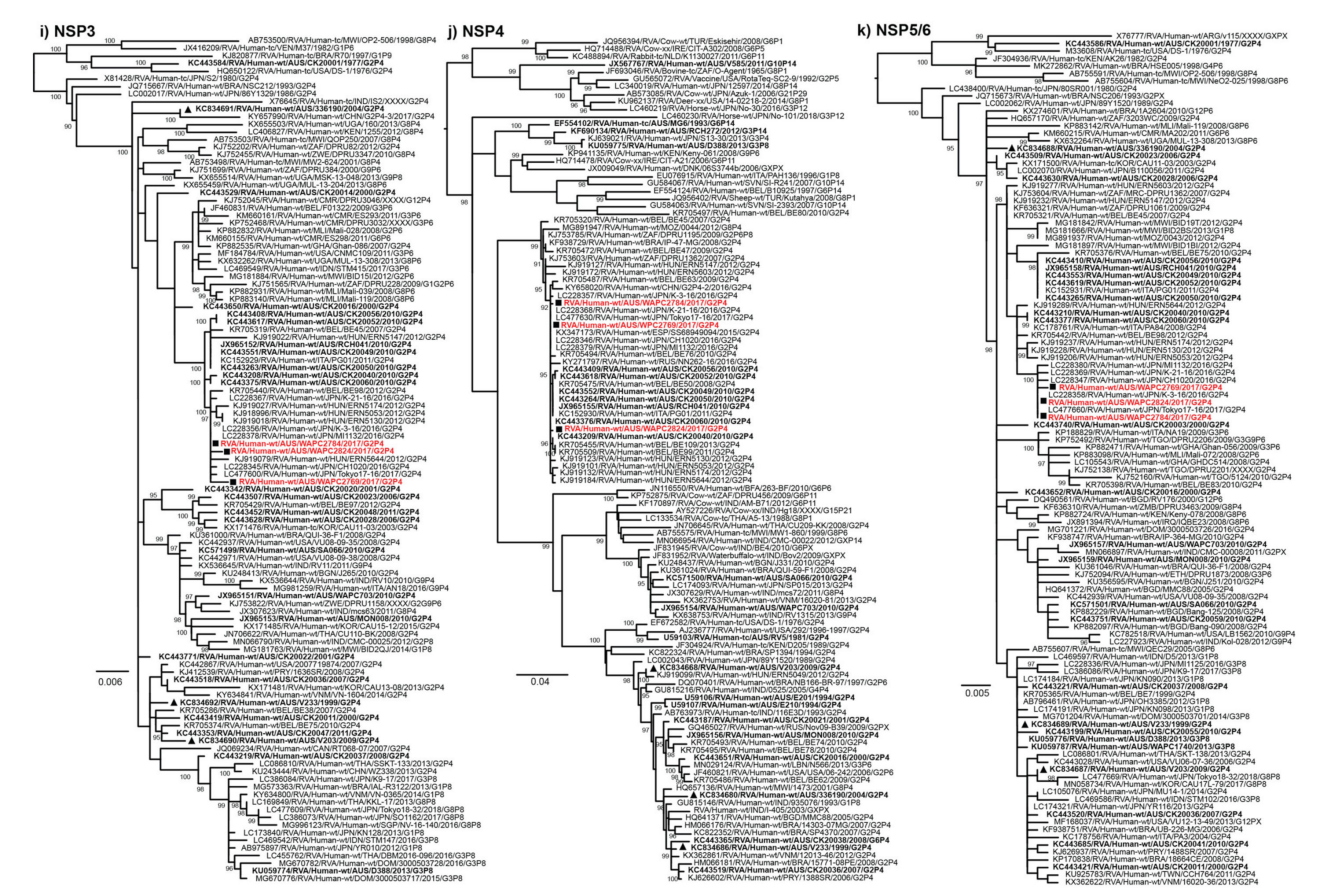
Maximum likelihood phylogenetic trees of (**a**) VP7, (**b**) VP4, (**c**) VP1, (**d**) VP2, (**e**) VP3, (**f**) VP6, (**g**) NSP1, (**h**) NSP2, (**i**) NSP3, (**j**) NSP4, and (**k**) NSP5/6 2017 Western Australia outbreak G2P[4] samples. The position of strains sequenced in this study are highlighted in red and with square symbols, previously characterised G2P[4] outbreak samples from the Northern Territory detected in 1999, 2004, and 2010 are denoted with triangle symbols. All Australian samples are in bold. Ultrafast bootstrap values ≥95% are shown.

Phylogenetic analysis of the 11 genome segments was conducted to investigate the genetic relationships of the three outbreak samples RVA/Human-wt/AUS/WAPC2769/2017/G2P[4], RVA/Human-wt/AUS/WAPC2784/2017/G2P[4], and RVA/Human-wt/AUS/WAPC2824/2017/G2P[4] to previously characterised Australian samples and global strains (Figure 5a–k). In the VP7 tree, the outbreak samples clustered with contemporary G2P[4] samples from Japan and Taiwan detected in 2016 and 2017 shared 99.74–100% nt and 99.24–100% aa similarity (Figure 5a). The outbreak samples did not cluster closely to previously characterised Australian samples. The most closely related were two samples from Victoria detected in 2010, sharing 99.47–99.61% nt and 99.62–100% aa similarity (Figure 5a). In the VP4 tree, the outbreak samples clustered with the same contemporary G2P[4] samples from Japan as in the VP7 tree and shared 99.61–99.83% nt and 99.48–100% aa similarity (Figure 5b). Again, the outbreak samples did not cluster closely to previously characterised Australian samples; most closely related to the same two samples from Victoria (RVA/Human-w/AUS/CK20040/2010/G2P[4] and RVA/Human-wt/AUS/CK20060/2010/G2P[4]) that shared 99.48–99.66% nt and 99.30–99.70% aa similarity (Figure 5b).

Across the VP1, VP2, VP3, and VP6 gene trees, the outbreak samples from this study continued to form conserved clusters with the contemporary Japanese samples RVA/Human-wt/JPN/MI1132/2016/G2P[4], RVA/Human-wt/JPN/K-21-16/2016/G2P[4], RVA/Human-wt/JPN/K-3-16/2016/G2P[4], RVA/Human-wt/JPN/Tokyo17-16/2017/G2P[4], and RVA/Human-wt/JPN/CH1020/2016/G2P[4] as observed in the VP7 and VP4 trees (Figure 5c–f). The samples RVA/Human-w/AUS/CK20040/2010/G2P[4] and RVA/Human-wt/AUS/CK20060/2010/G2P[4] were consistently the most closely related Australian samples to those from the 2017 outbreak. Across all trees, the 2017 outbreak samples fell within a clade that was comprised of a conserved group of G2P[4] strains from Belgium and Hungary that were detected in 2012, and the Australian samples RVA/Human-wt/AUS/CK20049/2010/G2P[4], RVA/Human-wt/AUS/CK20050/2010/G2P[4], RVA/Human-wt/AUS/CK20052/2010/G2P[4], RVA/Human-wt/AUS/CK20056/2010/G2P[4], and RVA/Human-wt/AUS/RCH041/2010/G2P[4].

Across the NSP1, NSP2, NSP3, NSP4, and NSP5 gene trees, the 2017 outbreak samples exhibited the same pattern across all trees: clustering with same group of contemporary Japanese samples, and falling within conserved clades comprised of G2P[4] strains from Belgium and Hungary that were detected in 2012, and Australian 2010 samples (Figure 5g–k).

The 2017 outbreak samples were not closely related to the samples RVA/Human-wt/AUS/V233/1999/G2P[4], RVA/Human-wt/AUS/336190/2004/G2P[4] and RVA/Human-wt/AUS/V203/2009/G2P[4], which were associated with prior outbreaks in the Northern Territory, often clustering in separate lineages or distinct clades. This suggests that the current outbreak variant was not derived from the prior G2P[4] outbreak variants that had undergone genetic drift or reassortment over the intervening years but were more closely related to a G2P[4] variant that has been detected in Japan, Hungary, and other regions of the world. It may be derived from the Australian 2010 G2P[4] variant that has undergone moderate genetic drift during global circulation.

### 2.6. Comparison of the Outbreak Samples to the G2 VP7 Gene Component of the RotaTeq Vaccine

**Figure 6:**
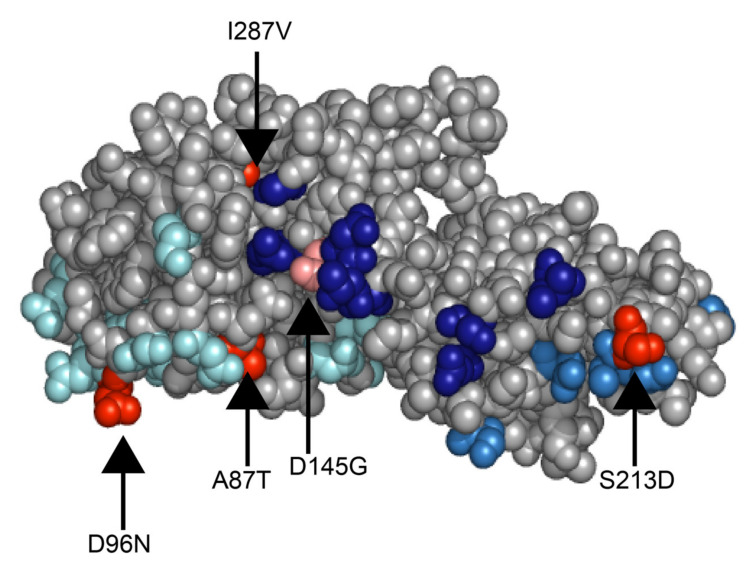
A surface representation of the VP7 monomer depicting the amino acid residues that differ between the 2017 G2P[4] WA outbreak samples and the G2 component of the RotaTeq vaccine strain (PDB ID: 3FMG). The antigenic epitopes are coloured as 7-1a in cyan, 7-1b in mid blue, and 7-2 in dark blue. The conserved residues that differ between the 2017 samples and the G2 component of the RotaTeq vaccine strain are shown in red and the residue that differed only in RVA/Human-wt/AUS/WAPC2784/2017/G2P[4] is shown in salmon.

The VP7 gene of the 2017 outbreak samples possessed 93.37–99.48% nt and 94.79–95.01% aa similarity with the G2 VP7 gene of RotaTeq. The amino acid differences between the outbreak samples and RotaTeq were analysed and 16 residues differed between the G2 component of RotaTeq and the two outbreak samples RVA/Human-wt/AUS/WAPC2824/2017/G2P[4] and RVA/Human-wt/AUS/WAPC2769/2017/G2P[4]. RVA/Human-wt/AUS/WAPC2784/2017/G2P[4] had 17 residues that differed. The altered residues that fell between amino acid 78 and 312 were mapped to the surface of the VP7 monomer to highlight mutations in proximity to the VP7 antigenic epitopes 7-1a, 7-1b, and 7-2 [25] (Figure 6). Mutations were observed in all three samples in antigenic epitope regions: positions A87T and D96N in antigenic region 7-1a, and S213D in region 7-1b. Additionally, the mutation D145G in the antigenic epitope region 7-2 was observed in RVA/Human-wt/AUS/WAPC2784/2017/G2P[4]. The three outbreak samples exhibited the residues D96N and S213D, which are amino acid changes that have been shown to escape neutralisation with monoclonal antibodies [26].

## 3. Discussion

Rotavirus was gazetted as a notifiable disease in WA in 2006 in part to monitor the effectiveness of the rotavirus vaccine when it was added to the childhood immunisation schedule in Australia in mid-2007 [11]. The introduction of rotavirus vaccines has lessened the once prominent seasonality of rotavirus infection in Australia [27,28]. Following campylobacteriosis and salmonellosis, rotavirus was the third most commonly notified enteric infection in the population of WA in 2017 [29]. A large increase in rotavirus notifications was noted in the second quarter of 2017, with the highest number of cases noted in May, indicating an outbreak occurring prior to the onset of winter (Figure 1).

A total of 236 rotavirus notifications were recorded in the second quarter of 2017, compared to the five-year second quarter average of 100.8 notifications, highlighting the scale of the outbreak. The five-year second quarter average was somewhat skewed by an outbreak in the second quarter of 2015 (Figure 1) that affected all WA regions, and predominantly affected non-Aboriginal people. Multiple outbreaks related to child care and aged care facilities were noted during this time, with the predominant strain identified as G12P[8] [30].

In contrast to 2015, the increase in the second quarter of 2017 was noted to disproportionally affect young Aboriginal children in the KIMB region, which is in the north of the state. A number of towns and Aboriginal communities in the KIMB region were affected. The KIMB PHU investigated the increase in notifications, with assistance from local government environmental health officers. Several public health interventions were implemented as a result of their investigations, including the distribution of a public health alert to local hospitals and Aboriginal medical service providers, liaising with environmental health officers and community health staff to provide public health advice for affected communities, and an interview on local radio.

It is noteworthy that an increased burden of rotavirus disease was reported elsewhere in Australia for 2017. Multiple outbreaks were recorded across Australia, due to equine-like G3P[8] in New South Wales and G8P[8] in New South Wales and Victoria [3]. In addition to the WA outbreak herein described, outbreaks due to G2P[4] were also reported in the Northern Territory (NT) and South Australia [3]. It is thought that the 2017 G2P[4] outbreak began in the NT and subsequently spread to rural and remote regions of WA adjacent to the border between these states [31]. A companion study described the weak protective effect of either Rotarix or RotaTeq vaccination in the setting of this outbreak [31]. Sub-optimal vaccine-effectiveness, particularly in the second year of life, has been reported in other high-burden, low-resource settings [32]. There are varied factors that could contribute to a reduced vaccine response, such as poor infant nutrition, the intestinal microbiota, co-morbid infections, as well as high levels of maternally derived anti-rotavirus antibodies [33].

The inclusion of rotavirus vaccines into the Australian NIP in 2007 has resulted in a considerable and sustained decrease in rotavirus morbidity across most of Australia, with a 71% decline in rotavirus-coded hospitalisations of children aged <5 years reported [34]. However, the observed decrease in hospitalisations has been less in Aboriginal and Torres Strait Islander children; they remain at greater risk of severe rotavirus disease requiring hospitalisation than their non-Indigenous counterparts [34]. Following rotavirus vaccine introduction in WA, significant declines in rotavirus-coded hospitalisation rates have been observed in all children aged <5 years, up to 79% among non-Aboriginal and up to 66% among Aboriginal children [35]. During the outbreak peak in May 2017 (122 cases), over a third of cases were hospitalised as a result of their infection, with Aboriginal people representing two thirds of these hospitalisations. As would be expected with rotavirus infection, the vast majority of cases hospitalised were ≤4 years of age, and Aboriginal children accounted for 71% of hospitalisations in this age group. Compared to their non-Indigenous counterparts, the paediatric Aboriginal population exhibit a greater burden of disease due to infections, and large, biannual rotavirus outbreaks have been reported in the Northern Territory [18,19,20,21,22]. Continued surveillance is critical to elucidate the complex factors that contribute to the occurrence of these outbreaks.

When the vaccination status of cases from the May peak was compared to hospitalisation status, vaccination did not appear to impact on whether a case was hospitalised. Fully or partially vaccinated children represented 75% of hospitalised cases (27/36) compared to unvaccinated eligible children accounting for 8% of hospitalised cases (3/36). Vaccination status was unknown for 3% of cases (1/36) and the remaining 14% of cases (5/36) were ineligible to have been vaccinated based on age. In May 2017, RotaTeq was the vaccine prescribed in the WA vaccination schedule and the vast majority of cases who were either fully or partially vaccinated were vaccinated with RotaTeq. Whilst a genotype-specific vaccine effectiveness has not been estimated for children in WA, the vaccine effectiveness of three doses of RotaTeq has been estimated at 82% (95% CI: 59–92) [36].

Full genome sequencing was performed on representative samples from the outbreak. These samples were found to be most closely related to Japanese G2P[4] strains detected in 2016 and 2017 across all genes in the genome. In one associated paper, these closely related samples were reported as minor G2P[4] variants circulating in the Mie prefecture in 2017 [37]. However, this variant was also detected in Tokyo in 2017, where G2P[4] was the dominant genotype, accounting for 40% of samples [38]. The outbreak samples also consistently clustered with Hungarian G2P[4] from 2012, where this genotype accounted for 13.5% of the samples genotyped in 2012 [39]. The WA outbreak samples clustered within a clade that also included G2P[4] strains from Australia that were circulating in 2010. These samples were collected during 2010–2011 when there was a substantial increase in G2P[4] strains in Australian states using the RotaTeq vaccine; G2P[4] strains replaced G1P[8] as the dominant genotype for the first time since vaccine introduction [23]. Overall, this suggests that the strain circulating during the 2017 WA outbreak is a global variant that was previously detected in Australia and has continued to be successfully transmitted in various regions around the world for over almost a decade. Based on the available sequencing, the majority of samples exhibit a relatively conserved genome that has not undergone substantial reassortment, with the diversity observed indicative of genetic drift over the years. It is highly likely that this variant represents a re-introduction into Australia rather than reflecting genetic drift that has only occurred in the Australian population. The 2017 variant was not closely related to G2P[4] strains that had caused prior outbreaks in the Northern Territory in 1999, 2004, and 2009 [18].

The VP7 gene of the 2017 WA outbreak samples was compared to the G2 VP7 gene component of the RotaTeq vaccine. A total of 16 residues differed between the G2 component of RotaTeq and the two outbreak samples RVA/Human-wt/AUS/WAPC2824/2017/G2P[4] and RVA/Human-wt/AUS/WAPC2769/2017/G2P[4], and RVA/Human-wt/AUS/WAPC2784/2017/G2P[4] had 17 residues that differed. However, this is not unexpected as the RotaTeq G2 VP7 gene is derived from a strain that was circulating in 1992; global strains have undergone extensive genetic drift over the intervening years. Three of these altered residues in all three outbreak samples were observed in antigenic epitopes at positions A87T and D96N in antigenic region 7-1a, and S213D in region 7-1b [25]. Altered residue D145G in region 7-2 was only observed in RVA/Human-wt/AUS/WAPC2784/2017/G2P[4]. Residues D96N and S213D have been shown to escape neutralisation with monoclonal antibodies [26]. The observed altered residues A87T, D96N, and S213D have been observed in the majority of G2P[4] strains circulating globally over the last two decades [40]. In particular, mutations A87T, D96N, D145G, and S213D were observed in G2P[4] strains associated with outbreaks in children in Indonesia in 2018 and a nosocomial outbreak in adults within a German hospital [41,42]. Genetic drift in VP7 antigenic epitope regions could adversely impact the effectiveness of the RotaTeq vaccine against G2P[4] strains. However, large-scale studies combining genetic and antigenic characteristics of circulating variants are required to further elucidate this. It is possible that genetic drift between circulating variants and the vaccine strain, in combination with host-related facts that impact vaccine effectiveness in this population contribute to the occurrence of these outbreaks.

A limitation of this study was that a stool sample was available for 70/122 cases from the May peak. Not genotyping all samples could result in the proportion of the different genotypes being over- or underestimated. However, it does not alter the result that G2P[4] was the dominant genotype in the KIMB region as 41/53 samples were available and genotyped. The 70 samples available for genotyping were representative of the age distribution of rotavirus cases in WA during this period. However, more samples from Aboriginal cases were genotyped compared to non-Aboriginal cases (76% vs. 45%) and this could have overestimated the proportion of G2P[4] cases reported. Similarly, more samples were genotyped from the remote areas of the KIMB and PILB regions, which may also have overestimated the proportion of G2P[4] cases seen. Given this study largely focuses on the KIMB region, it is unlikely that this had any major impact on the overall results of the study.

## 4. Materials and Methods

### 4.1. Notification Data

Data on WA cases of rotavirus were obtained from the WA Notifiable Infectious Disease Database (WANIDD). The notifications contained in WANIDD are received from medical practitioners and pathology laboratories under the provisions of the Public Health Act 2016 and subsequent amendments, and are retained in WANIDD if national case definitions are met. Rotavirus was listed as a notifiable disease in WA in July 2006 [11]. Data was extracted from WANIDD by optimal date of onset (ODOO) for the time period 01/01/2012 to 31/12/2017 and exported to Microsoft^®^ Excel 365 (Microsoft^®^, Version 1808, Redmond, WA, USA). The ODOO is a composite of the ‘true’ date of onset provided by the notifying doctor or obtained during case follow-up, the date of specimen collection for laboratory notified cases, and when neither of these dates is available, the date of notification by the doctor or laboratory, or the date of receipt of notification, whichever is earliest. Notification data are broken down by regions that are based on Public Health Unit (PHU) boundaries, reflecting WA Health administrative regions: Central/Wheatbelt (CENT), Goldfields (GOLD), Great Southern (GSTH), Kimberley (KIMB), Metropolitan Perth (METRO), Midwest (MIDW), Pilbara (PILB), and South West (STHW).

### 4.2. Vaccination Status

Records of vaccine administration were submitted to the Australian Immunisation Register (AIR) (curated by Services Australia, Australian Government). The AIR includes vaccines administered under the national immunisation program, school programs, and privately. CDCD staff accessed AIR to determine the rotavirus vaccine status of notified cases.

### 4.3. Rotavirus Positive Faecal Samples

A total of 122 faecal samples collected from children and adults presenting to hospital or general practice clinics with severe gastroenteritis in Western Australia during May, 2017 were determined to be rotavirus positive by a local diagnostic laboratory. Seventy de-identified rotavirus positive specimens were sent to the National Rotavirus Reference Centre (NRRC) at the Murdoch Children’s Research Institute. A further 27 samples did not have adequate remaining volume and were not sent for genotyping. There is no agreement with private pathology laboratories to forward samples for genotyping. As a result, 25/122 (20%) of samples were not genotyped. Where possible, metadata, including date of collection, date of birth, gender, and postcode, were collected. Samples were stored at −80 °C until analysis, allocated a unique laboratory code, and entered into a REDCap database.

### 4.4. Genotyping

Viral RNA was extracted from 10–20% (*w*/*v*) faecal extracts using the QIAamp Viral RNA mini extraction kit (QIAGEN, Hilden, Germany) according to the manufacturer’s instructions. Rotavirus G- and P-genotyping was performed using a hemi-nested multiplex RT-PCR assay [43]. First-round RT-PCR reactions were performed using the One Step RT-PCR kit (QIAGEN, Germany), using the VP7 (VP7F/VP7R), or the VP4 primer pair (VP4F/VP4R) [44,45]. The second-round genotyping PCR reactions were performed using the AmpliTaq^®^ DNA Polymerase with Buffer II (Applied Biosystems, Foster City, CA USA), together with specific oligonucleotide primers for G types (1, 2, 3, 4, 8, and 9) or P types ([4], [6], [8], [9], [10], and [11]) as previously described [4]. Gel electrophoresis of second-round PCR products was performed to determine the G- and P- genotype of each sample.

### 4.5. Conformation of Vaccine-Line Strains

Sequencing of VP6 and VP7 genes was performed for suspect RotaTeq samples with mixed G types or were P non-typeable as previously described [18].

### 4.6. Polyacrylamide Gel Electrophoresis

The 11 segments of rotavirus dsRNA were separated on 10% *w*/*v* polyacrylamide gel with 3% *w*/*v* polyacrylamide stacking gel at 25 mA for 16 h. The genome migration patterns (electropherotypes) were visualised by silver staining according to the established protocol [46].

### 4.7. Whole Genome Sequencing

Each of the 11 genes were reverse transcribed and amplified by PCR using the OneStep RT-PCR Kit (QIAGEN, Hilden, Germany) using gene-specific sense and antisense primers (primer sequences available upon request). RNA was denatured and reverse transcribed for 30 min at 45 °C, followed by PCR activation for 15 min at 95 °C. Then, 40 cycles of amplification for 10 s at 94 °C, 1 min at 55 °C, and 3 min at 68 °C, followed by a final extension for 10 min at 68 °C were performed. The amplicons were gel purified using the Wizard^®^ SV Gel and PCR Clean-Up System (Promega, Madison, WI, USA) according to the manufacturer’s instructions.

The purified products were pooled in equimolar concentrations and subjected to standard library construction for Illumina sequencing using the Nextera XT DNA Library Preparation Kit following the manufacturer’s recommendations for dual-indexed barcoding (Illumina Inc., San Diego, CA, USA). Normalised samples were pooled and sequenced using 500-cycle (2 × 250-bp paired-end) MiSeq reagent kits (v2; Illumina Inc., San Diego, CA, USA).

### 4.8. Sequence Assembly

Raw reads were trimmed for quality and adapters using BBDuk Adapter/Quality Trimming Version 38.37, duplicate reads were removed using Dedupe Duplicate Read Remover version 38.37 and pair-end reads were merged using BBMerge Paired Read Merger version 38.37, all performed within Geneious Prime. Reads were mapped to reference rotavirus genomes using the Bowtie2 mapper within Geneious Prime [47].

### 4.9. Assignment of Genotypes

The genotypes of each of the 11 genome segments were determined using the online RotaC v2.0 rotavirus genotyping tool (http://rotac.regatools.be, accessed on 18 January 2021) in accordance with the recommendations of the Rotavirus Classification Working Group (RCWG) [8].

### 4.10. Phylogenetic Analysis

Nucleotide similarity searches were performed using the BLAST server on the GenBank database at the National Center for Biotechnology Information, USA (www.ncbi.nlm.nih.gov, accessed on 18 January 2021). The nucleotide and amino acid sequences of each gene were compared with sequences available in the GenBank database that possessed the entire open reading frame. Multiple nucleotide and amino acid alignments were constructed using the Multiple Sequence Comparison by Log Expectation (MUSCLE) algorithm in Geneious Prime [48].

The best-fit nucleotide substitution model for each gene tree were tested and selected in IQTREE v1.6 using the using the Bayesian Information Criteria [49]. The selected nucleotide substitution models were GTR+F+R3 (VP1, VP3), GTR+F+G4 (VP4), TIM+F+G4 (NSP1, NSP2, NSP3), TIM+F+I+G4 (VP2) TN+F+G4 (VP6, NSP4), and HKY+F+G4 (VP7, NSP5/6). The maximum likelihood trees were inferred using IQTREE v1.6 with the robustness of branches assessed by 1000 bootstrap replicates using the ultrafast bootstrap feature [50]. The resulting trees were visualised and edited in FigTree v1.4.4 (http://tree.bio.ed.ac.uk/software/figtree/, accessed on 18 January 2021). Nucleotide and amino acid distance matrixes were calculated using the p-distance algorithm in MEGAX [51]. Structural analysis of the VP7 protein (PDB ID: 3FMG) was performed using the PyMOL Molecular Graphics System, Version 1.2r3pre (Schrödinger, Inc, New York, NY, USA).

### 4.11. Accession Numbers

The nucleotide sequences for genes described in this study have been deposited in GenBank under the accession numbers MW275246–MW275278.

## 5. Conclusions

This G2P[4] outbreak disproportionately impacted Aboriginal children ≤4 years of age in the remote Kimberley region of Western Australia. The G2P[4] variant circulating was closely related to contemporary Japanese G2P[4] samples, suggesting a global variant that exhibited the altered residues A87T, D96N, and S213D compared to the G2 component of the RotaTeq vaccine, residues that have been observed in the majority of G2P[4] strains circulating globally over the last two decades. Despite national vaccine coverage of 85.4%, outbreaks continue to occur in vaccinated populations in Australia, in particular impacting Aboriginal populations. These outbreaks pose particular challenges to regional areas and remote communities. Continued surveillance and characterisation of emerging variants are imperative to ensure the ongoing success of the rotavirus vaccination program in Australia.

## Figures and Tables

**Table 1:** Vaccination status of rotavirus cases from May 2017.

Vaccination Status					
Genotype	Full	Partial	Eligible But Not Vaccinated	Ineligible Due to Age ^1^	Total
G2P[4]	34	13	3	12	62
G3P[8]	1	0	0	2	3
G8P[8]	0	0	0	1	1
**Subtotal**	**35**	**13**	**3**	**15**	**66**
No data ^2^	13	16	2	21	52
**Total May cases**	**48**	**29**	**5**	**36**	**118**

^1^ Individuals ≥11 years of age were considered ineligible to have ever received a rotavirus vaccine dose based on age. ^2^ 52 samples were not sent to Murdoch Children’s Research Institute for genotyping.

**Table 2:** Genotype results for 70 rotavirus positive samples (ODOO* May, 2017).

Genotype	Region ^1^	Aboriginal	Non- Aboriginal	Total
G2P[4]	GOLD	1	3	4
	KIMB	36	4	40
	METRO	1	8	9
	MIDW		1	1
	PILB	3	8	11
	STHW		1	1
	**Total**	**41**	**25**	**66**
G3P[8]	METRO		3	3
G8P[8]	KIMB		1	1

^1^ Public Health Unit (PHU) boundaries, reflecting WA Health administrative regions: Goldfields (GOLD), Kimberley (KIMB), Metropolitan Perth (METRO), Midwest (MIDW), Pilbara (PILB), and South West (STHW). *OODO: Optimal date of onset.

## Data Availability

The nucleotide sequences for genes described in this study have been deposited in GenBank under the accession numbers MW275246-MW275278.
